# Ethnicity and suicide in England and Wales: a national linked cohort study

**DOI:** 10.1016/S2215-0366(24)00184-6

**Published:** 2024-08-01

**Authors:** Duleeka Knipe, Paul Moran, Laura D Howe, Saffron Karlsen, Nav Kapur, Lauren Revie, Ann John

**Affiliations:** Population Health Sciences, Bristol Medical School University of Bristol, Bristol, UK, South Asian Clinical Toxicology Research Collaboration, Faculty of Medicine, University of Peradeniya, Peradeniya, Sri Lanka; Population Health Sciences, Bristol Medical School University of Bristol, Bristol, UK; Population Health Sciences, Bristol Medical School University of Bristol, Bristol, UK, Medical Research Council Integrative Epidemiology Unit University of Bristol, Bristol, UK; Centre for the Study of Ethnicity and Citizenship, School of Sociology, Politics and International Studies University of Bristol, Bristol, UK; National Confidential Inquiry into Suicide and Safety in Mental Health, Centre for Mental Health and Safety, School of Health Sciences University of Manchester, Manchester, UK, National Institute for Health and Care Research Greater Manchester Patient Safety Research Collaboration University of Manchester, Manchester, UK, Mersey Care NHS Foundation Trust, Manchester, UK; Data and Analysis for Social Care and Health, Office for National Statistics, Newport, UK; Swansea University Medical School, Swansea University, Swansea, UK, Public Health Wales, Cardiff, UK

## Abstract

**Background:**

Understanding of ethnic disparities in suicide in England and Wales is poor as ethnicity is not recorded on death certificates. Using linked data, we examined variations, by sex, in suicide rates in England and Wales by ethnicity and migrant and descendant status.

**Methods:**

Using the Office for National Statistics 2012−19 mortality data linked to the 2011 census from the Public Health Research Database, we calculated the age-standardised suicide rates by sex for each of the 18 self-identified ethnicity groups in England and Wales. We present rates by age, sex, and methods used for suicide by ethnic group. We estimated age-adjusted and sex-adjusted incidence rate ratios (IRRs) using Poisson regression models for each minority ethnic group compared with the majority population. We involved people with lived experience in the research.

**Findings:**

Overall, 31 644 suicide deaths occurred over the study period, including 3602 (11%) in people from minority ethnic backgrounds, with a mean age of death of 43·3 years (SD 17·0, range 13−96). Almost all minority ethnic groups had a lower rate of suicide than the White British majority, apart from individuals who identified as being from a Mixed heritage background or White Gypsy or Irish Travellers. In females who identified as Mixed White and Caribbean, the suicide IRR was 1·79 (95% CI 1·45−2·21) compared with the White British majority; in those who identified as White Gypsy or Irish Travellers, the IRR was 2·26 (1·42−3·58). Rates in males identifying as from these two groups and those identifying as White Irish were similar to the White British majority. Compared with the non-migrant population, migrants had a lower rate of suicide regardless of ethnicity, but in the descendant population, people from a Mixed ethnicity background had a higher risk of suicide than the White British majority.

**Interpretation:**

There are ethnic disparities in suicide mortality in England and Wales, but the reasons for this are unclear. The higher rate in previously overlooked minority ethnic groups warrants further attention.

**Funding:**

Wellcome Trust.

## Introduction

Suicide is a leading cause of premature mortality worldwide,^1^ with more than 6500 people dying by suicide in the UK annually.^2–4^ In England and Wales, ethnic disparities in suicide mortality have been reported, with high rates of suicide observed in individuals who self-identify as White or having a Mixed ethnic heritage in both sexes, and some evidence that rates in these groups are increasing over time.^5–7^ This risk does not appear to be explained by socioeconomic differences.^7^

Ethnicity is currently not recorded on death certificates. Poor quality ethnicity data are not restricted to the UK—these data are also not routinely collected in other European countries. Previous investigations in the UK have relied on country of birth information or names as proxies for ethnicity.^8^ In 2021, the Public Health Research Database (PHRD) was released, which provided mortality data linked to self-reported ethnicity data from the 2011 census. Although analyses using these data have provided important insights into ethnic disparities in suicide, they remain limited.^5–7^ This previous work combined White minority ethnic groups (Gypsy, Irish Traveller, Irish, and Other) with the White British majority, did not comprehensively examine suicide rates in all ethnicity groups, and did not explore method of suicide. Restricting access to methods of suicide is a key suicide prevention strategy, so understanding ethnic differences in method choice is important for prevention priorities in specific localities and internationally. Additionally, despite previous research indicating a differing risk of suicide in migrants versus their descendants,^9,10^ previous investigations considered these two groups as a single entity.

There are several reasons why addressing this evidence gap is important. More than 15 million people in England and Wales self-identify as having a minority ethnic background—a figure that has increased by more than 20% over the past decade.^11^ Two-thirds of individuals with a minority ethnic background were born outside of the UK. Moreover, individuals from a minority ethnic group experience more risk factors for suicide (eg, employment difficulties, deprivation, and discrimination) than the White British majority population. In addition, migrant populations can face uniquely severe social and psychological stressors (eg, pre-migration trauma and loss of social contacts) that can predispose them to poor mental health and suicidal behaviour.^12^ The impact of these stressors is likely to differ by gender, age, and whether people are migrants or descendants of migrants. Yet, our understanding of ethnic disparities in suicide mortality in the UK is still poor.

To address these gaps, we used data from the PHRD to calculate suicide rates in England and Wales by ethnicity and migrant or descendant status in males and females, and to assess whether the rate of suicide in minority groups differed compared with the ethnic majority. Additionally, we calculated sex ratios and age-specific rates by ethnicity and sex, and determined the most common method of suicide by ethnic group stratified by sex.

## Methods

### Study design and participants

We conducted a national linked cohort study using the Office of National Statistics (ONS) PHRD, which has linked death registrations in England and Wales to 2011 census data using National Health Service (NHS) numbers. Every individual registered with a general practice is assigned a unique NHS number; in the PHRD, 95% of census-enumerated individuals were linked to their NHS number.^5^ All deaths that occurred between Jan 1, 2012 and March 31, 2019 were included in this analysis, with their data linked to the 2011 census (usual residents only). Our analysis accounts for 97% of all deaths that occurred during this time period.^5,13^ The resulting data account for 90% of the total population enumerated in the census.^5^ Ethical approval was granted by the National Statisticians Data Ethical Advisory Committee.

The lead author (DK) identifies as being from a racially minoritised background and has been bereaved by suicide. She was involved in all aspects of this work and additional individuals with lived experience across the UK from racially minoritised backgrounds were involved, through the charitable mental health organisations Nilaari Agency and Taraki, in the interpretation and dissemination of findings.

### Procedures

In the 2011 census, individuals were asked “what is your ethnic group?”. Respondents were able to select from 18 response categories (table 1).^14^ Respondents were also asked “what is your country of birth?” with six response options: England, Wales, Scotland, Northern Ireland, Republic of Ireland, or elsewhere. If respondents selected elsewhere, they were asked to write the current name of the country in which they were born.

The PHRD data exclude individuals who entered the UK in the year before the census (due to the high likelihood that they might have left during the study period) and those older than 100 years at the time of the census. There are ethnic differences in linkage rates, which were lower for those identifying as being from a Black other or other ethnic group, and those who died in England and Wales but who were born in central and western Africa. Further details are provided by Nafilyan and colleagues.^13^

Following census−mortality linkage, the PHRD only contains data related to deaths. We used population counts by ethnicity, country of birth, age, and sex to generate a cohort dataset. We then subtracted the number of people who died by any cause before the end of the follow-up period (ie, March 31, 2019) in each relevant strata in the population data. We then expanded the frequency tables into a cohort of individuals who were alive during the 2011 census and were not recorded to have died, by any cause, during our follow-up period. We appended the single record death data to this cohort file.

### Statistical analysis

We used survival models to estimate the rate of suicide and to calculate incidence rate ratios (IRRs). Follow-up time for individuals was calculated from Jan 1, 2012 until the date of death or the end of the follow-up period (March 31, 2019). Consistent with ONS definitions, we identified all suicide deaths as ICD-10 codes X60−X84/X600−X849 for those aged 10 years and older, and all undetermined intent deaths were included in suicide counts for those older than 15 years (Y10−Y34/Y100−Y349).

Our analysis was split into investigations focused on ethnicity, migration, and descendant status. For all investigations we calculated sex ratios, age-standardised suicide rates per 100 000 person-years by sex, and IRRs using Poisson regression models adjusting for age and sex. We used the age at the start of follow-up (ie, census enumeration: March 27, 2011). For all comparisons, the White British majority group was used as the base category.

For the investigation into ethnicity, we maintained the 18 census ethnicity categories. When counts fell below five, we do not present these data, in keeping with ONS permissions for data use (appendix p 2). We also removed them from the overall counts due to risk of identification of the individual. We applied further secondary suppression to data cells to avoid statistical disclosure. We present suicide rates by broad age groups (10−24 years, 25−64 years, and ≥65 years) defined to represent adolescence and emerging adulthood,^15^ working age adults, and older adults. The distributions of methods used for suicide are also presented by sex and ethnicity grouping. For the migrant investigation, we categorised individuals who might be migrants by identifying non-UK born individuals (ie, using country of birth data) and grouped them by six broader ethnicity categories. The broader categories are the ones used by the census. For this analysis, we opted to collapse the ethnicity groups to avoid unintended disclosure and to maximise the power of our analysis. We compared migrant populations with individuals who were born in the UK regardless of ethnicity status (those who identified as White British but were born outside of the UK were excluded in this analysis). Lastly, for our investigation into descendant status, we identified individuals who were non-migrants but probably descendants of migrants. We categorised individuals as descendants if they were born in the UK, but they identified as non-White British. Again, we used the six ethnicity categories for this analysis. These descendants are typically who would be referred to as second-generation migrants and are probably British, and therefore we used the UK-born White British population as the reference category.

### Role of the funding source

The funder of the study had no role in study design, data collection, data analysis, data interpretation, or writing of the report.

## Results

All deaths that occurred between Jan 1, 2012 and March 31, 2019 (n=3 753 799) were included in this analysis. During the 63-month follow-up period there were 31 644 suicide deaths (23 517 males and 8127 females), including deaths recorded as being of undetermined intent in individuals aged 15 years and older (n=5695; 18%). Overall, 3602 (11%) of 31 644 suicide deaths were in individuals with a minority ethnic background, including 2601 (72·2%) males and 1001 (27·8%) females. The mean age of death in individuals from a minority ethnic background was 43·3 years (SD 17·0, range 13−96), and in the White British group it was 49·9 years (SD 17·8, range 11−102).

There was considerable variation in the rates of suicide by ethnicity and sex (table 1), with rates ranging between 1·61 and 21·12 per 100 000 person-years in males and 0·80 and 8·65 per 100 000 person-years in females. The point estimates for many of the minority ethnic groups were imprecise, as reflected by the wide CIs. More males than females died by suicide overall, but this difference varied by ethnic group: in individuals who identified as

Black other, there was a sex rate ratio of 9:2 (male to female); in individuals who identified as Arab, there was no variation between males and females. In males, the rate of suicide was lower (IRR 0·16−0·67) in all minority ethnic groups than in the White British majority, except for the four Mixed ethnicity and the White Gypsy, White Irish, or Irish Traveller groups, who appeared to have a similar rate of suicide to the White British majority. In females, the rate of suicide was lower (IRR 0·23−0·79) in most minority ethnic groups compared with their majority counterparts. The rates of suicide in females who identified as Chinese (IRR 0·82 [95% CI 0·63−1·07]), Mixed White and Asian (1·32 [0·99−1·76]), Mixed White and Black African (0·90 [0·55−1·47]), and Mixed other (0·83 [0·58−1·19]) were statistically consistent with the White British majority group. In females who identified as Mixed White and Caribbean, the rate of suicide was higher (IRR 1·79 [1·45−2·21]) than the White British majority, and the rate in females who identified as White Gypsy or Irish Traveller was more than double that of the White majority (2·26 [1·42−3·58]).

When sufficient data were available, we calculated age-group and sex-specific suicide rates (tables 2, 3). In males, the rate of suicide varied considerably by age and ethnic group (table 2). Older males from a Chinese ethnic background had an elevated rate of suicide compared with their younger counterparts (13·15 *vs* 2·41−4·53 per 100 000 person-years). Working-aged males who identified as White British had a higher rate of suicide than their younger and older equivalents, a pattern also observed in males who identified as White Gypsy or Irish Travellers. The rate of female suicide was broadly similar across age groups by ethnicity, with the exception of older females who identified as Chinese, who had a rate five times higher (16·96 *vs* 3·13−3·26 per 100 000 person-years) than their younger counterparts (table 3). This elevated rate in older adults was also observed in females identifying as Black African (5·45 *vs* 1·72−2·01 per 100 000 person-years) and Other (10·93 *vs* 2·00 per 100 000 person-years). The rate of suicide also appears to be much higher in working age females from a Mixed White and Caribbean background compared with adolescents and emerging adults (11·18 *vs* 5·27 per 100 000 person-years).

The greatest proportion of suicide deaths were by hanging in males and females across all ethnic groups (appendix p 3). For example, hanging was the method used by 57·8% of males and 47·8% of females who identified as Indian.

Migrants constituted 2211 (7%) of 30 820 suicide deaths in the sample (1576 males; 635 females). The rate of suicide was consistently lower among migrants than non-migrants, regardless of ethnicity (table 4). The lower rate of suicides in migrants was similar in term of magnitude of effect in both sexes. The male to female ratio varied considerably between ethnic groups, but generally more males died by suicide than females (with a ratio of 6·3 for migrants who identified as Asian). Female migrants from the other ethnic groups appeared to be just as likely to die by suicide as their male counterparts.

1474 (5%) of 28 609 suicide deaths in individuals who were born in the UK occurred in those who did not identify as White British (1050 males; 424 females). The rate of suicide in males was lower (roughly half) in migrant descendants who identified as Asian, Black, or Other than those who identified as White British, but rates were similar for migrant descendants who identified as being from a Mixed or White other ethnicity (table 4). In females, suicide rates were lower in migrant descendants who identified as Asian or Black than the White British majority. Similar rates to the majority population were observed in female descendants who identified as White other or Other, with a 63% (IRR 1·63 [95% CI 1·39−1·90]) higher rate observed in female descendants who identified as from a Mixed ethnicity than the majority population. The male to female ratio in migrant descendants consistently indicated an elevated rate of suicide in males compared with females, with the greatest difference observed in descendants who identified as Black.

## Discussion

In England and Wales, we found that almost all minority ethnic groups have a lower rate of suicide than the White British majority, with the exception of individuals who identify as being from a Mixed heritage background or White Gypsy or Irish Travellers. In males, the rate of suicide in these two groups and White Irish individuals was similar to the White British majority, but in females, rates were 79% higher in individuals from a Mixed White and Caribbean heritage background, and more than double in White Gypsy or Irish Travellers. The higher rate of suicide seen in some minority ethnic groups appears to be driven by a greater suicide risk in descendant populations (ie, potentially minority ethnic British-born individuals), rather than migrants. More males than females died by suicide across all ethnic groups, with considerably higher rates in males who identified as Black other compared with females (9:1). Older individuals (aged ≥65 years) from a Chinese ethnic background had a higher rate of suicide than their younger counterparts. There were no apparent differences in methods used for suicide across minority ethnic groups.

The lower risk of suicide in most minority ethnic groups compared with the White British majority analysed here is consistent with evidence from across the world.^8^ This lower rate of suicide might reflect a genuinely lower risk in these demographic groups, or it could reflect differences in the classification of suicide. A genuinely lower rate of suicide might be attributed to the effect of stronger religious beliefs among individuals from minority ethnic groups,^16,17^ or greater community cohesion and support through extended family networks. It is notable that the influence of these protective factors would appear strong enough to mitigate other factors that disproportionately affect individuals in minority ethnic groups living in the UK, which are generally considered to increase risks of suicide, including experiences of childhood adversity, financial difficulty, severe mental illness, discrimination, and a lower likelihood of receiving effective treatment for mental health problems.^1,18,19^ An alternative explanation relates to research identifying a greater risk of the misclassification of suicide involving people in minority ethnic groups. There is evidence to suggest that the attribution of suicide at inquest differs by ethnicity from a single coroner study in London, England: individuals perceived as being non-White were more likely to have their suicide death misclassified as a non-suicide (eg, accidental or misadventure).^20^ Other studies have not confirmed this finding. It might be that different ethnic densities in different areas affect results, but this has not yet been shown in any study. Our initial exploration of misclassification of suicide deaths through our analysis of accidental hanging found a greater chance of minority ethnic individuals having their deaths classified as accidental (unpublished). However, the analysis was underpowered for more granular investigation by ethnicity. Further work on misclassification using this method is unlikely to definitively answer questions relating to misclassification because of limitations of the ONS mortality data.

The elevated suicide risk among individuals with a Mixed ethnicity background compared with the ethnic majority, identified here, confirms previous findings^6,7^ and extends them to provide granular data by Mixed ethnicity and age and sex. Individuals who are from a Mixed ethnicity background are more likely to be socially assigned^10^ and pass as White than other minority ethnic groups.^21,22^ Ethnic identities can be differentiated as internal (what we believe about ourselves), expressed (what we say we are), and external (what others assume we are).^23^ For many people from racially minoritised backgrounds, their ethnic identity forms a strong part of their overall identity. For individuals from Mixed heritage backgrounds, the sense of ethnic identity can be fluid over time and in different contexts—what is internally identified and expressed can change at different points in time and in different social situations.^24^ Individuals from a Mixed heritage background therefore can experience liminality, the concept of being in-between in terms of their ethnic identity. Individuals from a Mixed ethnic background also experience identity invalidation, whereby their internal and expressed ethnic identities are not supported by others. There is evidence that identity invalidation occurs to a greater degree in those from a Mixed heritage background in England compared with other ethnic groups.^25^ These experiences of liminality and invalidation can be distressing and have been shown to be associated with suicidal ideation and attempts.^26^ Health interventions targeting this group of individuals are scarce, despite this demographic population growing rapidly in several countries around the world. This elevated risk requires further investigation to develop appropriate strategies to support these individuals. Interventions could be targeted at supporting parents, ensuring more inclusive school environments, and services that acknowledge the particular challenges individuals from this demographic group face. Individuals from a Mixed heritage background are currently being overlooked—current strategies to support individuals from minority ethnic backgrounds are focused on monoethnic groups. These strategies might not be appropriate nor effective in supporting individuals who identify as having multiple ethnicities.

We also observed a higher rate of suicide in individuals who identified as being White Gypsy or Irish Travellers. Evidence from Ireland shows a rate of suicide among male Travellers of nearly 7 times greater than that of the general population (with no statistical evidence of an elevated risk among the female Traveller group),^27^ much higher than in our findings. This discrepancy might be explained by differences in the experiences of Irish Travellers in England and Wales compared with those in Ireland, or it could be due to methodological differences between the studies.^27^ The reasons for the elevated risk of suicide in this population probably relate to higher rates of mental illness, higher rates of morbidity, long-term health conditions combined with delayed help-seeking and poor access to care, lower educational attainment, insecure accommodation, and economic exclusion.^28^ These risk factors are more prevalent in this group compared with any other ethnic group,^29^ and these areas are key targets for culturally sensitive co-designed future interventions.

Migrants appeared to have a lower rate of suicide than the non-migrant population, regardless of ethnicity. The lower risk of suicide is consistent with the wider observed migrant mortality advantage,^30^ and it is supportive of several hypotheses in migrant health. The first is the so-called healthy migrant hypothesis—ie, those who have a health advantage over others in their country of origin are more likely to migrate, and, for a period of time, they also have a health advantage over the host population.^31^ The second hypothesis is the so-called salmon bias hypothesis, which speculates that the lower mortality rate observed in migrant populations is explained by the fact that migrants might return to their countries of origin during the follow-up period and later die there. Under such circumstances, their death would not be counted in the mortality data in the host country, but they would contribute to the original denominator. This hypothesis certainly could be the reason for the lower rate of suicide in migrant populations, as between 2011 and 2018 the rate of emigration of non-British citizens increased by more than 30%.^32^ The final hypothesis works in a similar way to the salmon bias hypothesis. Those who develop ill health (in this case, poor mental health) are more likely to return to their countries of origin—the so-called unhealthy remigration hypothesis.^30^ Additionally, selection bias might play a part in the apparent lower risk. The migrant group captured within this dataset are those who were living in England and Wales for at least a year before the census, reflecting a more settled migrant population that probably excludes those (eg, sanctuary seekers) who are at a higher risk of suicide.^33^ Additionally, the census is unlikely to capture irregular migrants (eg, those who entered on irregular modes of entry, or those who overstay their visas), a migrant population who might have a higher risk of suicide.

Previous work in the UK has not investigated the risk of suicide in migrant and descendant populations separately. Our findings of an elevated risk of suicide in descendants but not migrant populations is consistent with the four existing international studies,^8^ except for a study from Norway, where a lower risk was observed in the migrant descendant group.^34^ However, the authors of this study urge caution in the interpretation of this estimate, as they suggest that this group is poorly defined in their dataset.

This study has methodological limitations that need to be considered when interpreting the findings. First, the PHRD is likely to be affected by selection bias. People from minority ethnic backgrounds are less likely to take part in the census. To be included in the PHRD, individuals needed to have been registered with a general practitioner. Some minority ethnic populations are likely to experience barriers registering with a general practitioner,^35^ so are potentially at higher risk of suicide (ie, those without access to health services) and more likely to be excluded from the dataset than other populations. Second, as described previously, the migration population included in this dataset is only a subset of all migrants. The dataset is likely to exclude recent migrants and irregular migrants. Additionally, the migrant profile captured within this dataset (2011−19) is likely to be different to the contemporaneous migrant population. Third, although we attempted to maintain the granularity of ethnicity categorisation available within the PHRD, we were limited by the relatively low numbers of suicide deaths in some minority ethnic groups. In these instances, we have either had to collapse groups into broader categories or exclude them from our analysis altogether. All the assumptions in creating and aggregating categories will probably have introduced biases, including the exclusion of those who identified as White British but were born outside of the UK. Lastly, coronial misclassification of suicide does occur,^36^ and misclassification rates might differ by ethnicity.^20^ It is possible that the lower rate of suicide observed in some groups is due to bias in the ascertainment of the outcome.

This study presents a paradoxical finding (given the greater clustering of risk factors in this group) of a lower rate of suicide in almost all minority ethnic groups compared with the White British majority, with the exception of individuals from a Mixed ethnicity or White Gypsy or Irish Traveller backgrounds. Better understanding is needed of the reasons for differences in suicide rates by ethnicity. It might be that groups with higher rates of suicide face greater socioeconomic adversity, which has been shown to be associated with greater suicide risk in this dataset.^6^ The differential patterning of suicide by ethnic group suggests that a single prevention approach is unlikely to be effective for all. The availability of census-linked suicide mortality data has helped shine a light on previously overlooked demographic groups. We now need to better understand the drivers of these ethnic disparities in suicide risk to inform effective future suicide prevention strategies.

## Supplementary Material

Supplementary appendix

## Figures and Tables

**Table 1:** Suicide rates and IRRs by self-reported ethnicity and sex in England and Wales

	Male			Female			Male to Female ratio
	Suicide (n)	ASR (95% CI)	IRR (95% CI)	Suicide (n)	ASR (95% CI)	IRR (95% CI)	
Asian							
Bangladeshi	37	1·61 (0.64·5−03)	0·20 (0·14−0·27)	14	1·05 (0·21−5·61)	0·27 (0·16−0·44)	1·53
Chinese	51	6·85 (2·12−26·87)	0·29 (0·22−0·38)	54	5·58 (2·07−16·92)	0·82 (0·63−1·07)	1·24
Indian	350	7·38 (4·71−12·91)	0·51 (0·46−0·56)	137	3·08 (1·52−7·03)	0·63 (0·53−0·75)	2·40
Pakistani	165	2·97 (1·47−6·89)	0·25 (0·21−0·30)	64	1·11 (0·38−3·68)	0·49 (0·39−0·63)	2·68
Other	114	6·50 (3·36−14·76)	0·44 (0·38−0·51)	38	2·18 (0·86−6·52)	0·27 (0·19−0·36)	2·98
Black							
African	129	3·73 (1·91−8·78)	0·32 (0·27−0·38)	57	2·33 (0·80−7·53)	0·39 (0·30−0·51)	1·60
Caribbean other	145	7·27 (3·89−14·3)	0·55 (0·47−0·65)	53	2·26 (0·91−6·23)	0·55 (0·42−0·72)	3·22
Other	43	7·34 (2·00−36·01)	0·40 (0·29−0·53)	7	0·80 (0·16−4·24)	0·28 (0·15−0·52)	9·18
Mixed							
White Asian	108	12·21 (5·65−32·60)	0·94 (0·78−1·14)	44	6·45 (2·04−23·99)	1·32 (0·99−1·76)	1·89
White Black African	44	14·44 (4·75−52·67)	0·89 (0·67−1·18)	16	3·29 (0·77−16·08)	0·90 (0·55−1·47)	4·39
White Caribbean	148	14·12 (7·16−31·34)	1·04 (0·88−1·21)	86	7·35 (3·21−22·48)	1·79 (1·45−2·21)	1·92
Other	70	10·04 (4·00−32·11)	0·73 (0·58−0·92)	29	3·77 (1·31−13·38)	0·83 (0·58−1·19)	2·66
White							
British	20 774	13·72 (12·99−14·50)	1	7014	4·41 (4·03−4·84)	1	3·11
Gypsy or Irish Traveller	31	21·12 (6·53−86·05)	1·40 (0·99−1·96)	14	8·65 (2·67−30·71)	2·26 (1·42−3·58)	2·44
Irish	285	14·36 (9·14−24·05)	1·09 (0·97−1·22)	68	3·37 (1·45−9·36)	0·78 (0·61−0·98)	4·20
Other	792	11·27 (8·02−16·29)	0·67 (0·62−0·72)	294	4·25 (2·62−7·13)	0·76 (0·68−0·85)	2·66
Arab	19	1·94 (0·52−7·81)	0·16 (0·10−0·25)	6	2·13 (0·45−10·89)	0·23 (0·10−0·52)	0·91
Other	70	7·43 (2·79−22·66)	0·40 (0·32−0·51)	20	2·87 (0·94−9·46)	0·44 (0·29−0·68)	2·59

ASR=age-standardised rate. IRR=incidence rate ratio.

**Table 2:** Male suicide rates by ethnicity and age group

	Suicide (n)			Suicide rate per 100 000 (95% CI)		
	Age 10−24 years	Age 25−64 years	Age ≥65 years	Age 10−24 years	Age 25−64 years	Age ≥65 years
Asian							
Bangladeshi	21	16	<5	4·42 (2·88−6·78)	2·11 (1·29−3·45)	··
Chinese	11	32	8	2·41 (1·34−4·36)	4·53 (3·20−6·40)	13·15 (6·58−26·30)
Indian	64	256	30	6·02 (4·71−7·70)	8·36 (7·39−9·45)	8·48 (5·93−12·13)
Pakistani	42	72	<5	3·83 (2·83−5·18)	3·67 (2·91−4·63)	··
Other	49	106	10	7·01 (5·30−9·28)	6·41 (5·30−7·75)	8·69 (4·68−16·15)
Black							
African	57	72	<5	6·58 (5·08−8·53)	4·01 (3·18−5·05)	··
Caribbean other	24	108	13	5·87 (3·93−8·75)	9·94 (8·23−12·01)	5·40 (3·13−9·29)
Other	9	34	<5	3·32 (1·73−6·39)	7·46 (5·33−10·44)	··
Mixed							
White Asian	42	66	<5	10·59 (7·83−14·33)	15·94 (12·52−20·28)	··
White Black African	18	26	<5	10·95 (6·90−17·38)	12·79 (8·71−18·78)	··
White Caribbean	60	88	<5	10·60 (8·23−13·66)	18·69 (15·16−23·03)	··
Other	26	44	<5	9·08 (6·18−13·33)	11·35 (8·45−15·26)	··
White							
British	3092	14 736	2946	10·25 (9·89−10·62)	17·54 (17·26−17·83)	12·75 (12·3−13·22)
Gypsy or Irish Traveller	5	26	<5	8·73 (3·64−20·98)	27·80 (18·93−40·83)	··
Irish	16	197	72	9·86 (6·04−16·10)	18·06 (15·71−20·77)	16·90 (13·42−21·29)
Other	123	615	54	8·75 (7·33−10·44)	10·85 (10·03−11·74)	13·83 (10·59−18·05)
Arab	6	13	<5	2·62 (1·18−5·84)	2·45 (1·42−4·22)	··
Other	16	46	8	5·94 (3·64−9·70)	5·79 (4·34−7·73)	14·50 (7·25−28·99)

Cell counts less than 5 are not presented and secondary suppression has been applied to other cells to avoid statistical disclosure.

**Table 3:** Female suicide rates by ethnicity and age group

	Suicide (n)			Suicide rate per 100 000 (95% CI)		
	Age 10−24 years	Age 25−64 years	Age ≥65 years	Age 10−24 years	Age 25−64 years	Age ≥65 years
Asian						
Bangladeshi	5	9	<5	1·11 (0·46−2·67)	1·30 (0·67−2·49)	··
Chinese	15	27	12	3·13 (1·89−5·20)	3·26 (2·23−4·75)	16·96 (9·63−29·86)
Indian	23	101	13	2·43 (1·62−3·66)	3·38 (2·78−4·11)	3·29 (1·91−5·66)
Pakistani	11	27	<5	1·07 (0·59−1·94)	1.44 (0·99−2·10)	··
Other	14	50	<5	2·24 (1·33−3·78)	2·65 (2·01−3·50)	··
Black						
African	18	34	5	2·01 (1·26−3·19)	1·72 (1·23−2·41)	5·45 (2·27−13·09)
Caribbean other	19	34	<5	4·50 (2·87−7·05)	2·55 (1·83−3·57)	··
Other	<5	7	<5	··	1·54 (0·73−3·23)	··
Mixed						
White Asian	20	24	<5	5·43 (3·51−8·42)	6·17 (4·13−9·20)	··
White Black African	9	7	<5	5·53 (2·88−10·64)	3·33 (1·59−6·98)	··
White Caribbean	30	56	<5	5·27 (3·68−7·54)	11·18 (8·61−14·53)	··
Other	7	22	<5	2·36 (1·13−4·96)	5·03 (3·31−7·64)	··
White						
British	922	4916	1176	3·18 (2·98−3·39)	5·77 (5·61−5·93)	4·03 (3·80−4·26)
Gypsy or Irish Traveller	<5	14	<5	··	14·03 (8·31−23·68)	··
Irish	<5	48	20	··	4·42 (3·33−5·86)	3·42 (2·20−5·30)
Other	49	245	34	3·14 (2·37−4·15)	3·92 (3·46−4·44)	5·67 (4·05−7·93)
Arab	<5	<5	<5	··	··	··
Other	<5	13	7	··	2·00 (1·16−3·45)	10·93 (5·21−22·93)

Cell counts less than 5 are not presented and secondary suppression has been applied to other cells to avoid statistical disclosure.

**Table 4:** Suicide rates and IRRs by migratory−descendant status, and sex

	Male			Female			Male to female ratio
	Suicide (n)	ASR (95% CI)	IRR (95% CI)	Suicide (n)	ASR (95% CI)	IRR (95% CI)	
**Non-migrant (UK born)**							
Asian	314	7·22 (3·70−24·39)	0·54 (0·49·0·61)	118	2·45 (1·17·7·16)	0·65 (0·54·0·79)	2·95
Black	157	11·47 (4·07−50·12)	0·54 (0·46−0·63)	65	1·85 (0·88−3·98)	0·65 (0·51−0·83)	6·16
Mixed	322	14·64 (8·71−30·88)	1·08 (0·96−1·20)	160	8·00 (3·77−21·88)	1·63 (1·39−1·90)	1·83
White British*	20 324	13·72 (12·98−14·51)	1	6811	4·39 (4·00−4·82)	1	3·12
White other	231	14·55 (8·58−27·15)	1·05 (0·92−1·20)	74	4·65 (2·07−11·83)	1·13 (0·90−1·42)	3·12
Other	26	8·12 (2·28−35·29)	0·51 (0·35−0·75)	7	5·73 (1·07−33·16)	0·50 (0·24−1·04)	1·42
**Migrant (not UK born)**							
Asian	407	5·00 (3·27−8·13)	0·31 (0·28−0·34)	193	0·80 (0·16−4·24)	0·44 (0·38−0·51)	6·25
Black	164	4·54 (2·49−8·83)	0·34 (0·29−0·40)	58	6·45 (2·04−23·99)	0·32 (0·25−0·41)	0·70
Mixed	63	7·69 (3·01−20·77)	0·55 (0·43−0·70)	21	3·29 (0·77−16·08)	0·54 (0·35−0·82)	2·34
Non-migrantt	21 374	13·5 (12·79−14·27)	1	7235	7·35 (3·21−22·48)	1	1·84
White other	879	11·77 (8·76−16·01)	0·71 (0·67−0·76)	343	3·77 (1·31−13·39)	0·74 (0·67−0·83)	3·10
Other	63	4·97 (1·80−15·61)	0·26 (0·21−0·34)	20	4·41 (4·03−4·84)	0·34 (0·22−0·53)	1·13

ASR=age-standardised rate. IRR=incidence rate ratio. *Excludes individuals who identify as White British but were born outside of the UK. †Includes those born in the UK regardless of ethnicity.

## Data Availability

The ONS PHRD is available on the ONS Secure Research Service for accredited researchers. Researchers can apply for accreditation through the Research Accreditation Service.

## Research in context

### Evidence before this study

Suicide is a major public health problem, with social exclusion being a risk factor for suicide. The UK is one of the top five destinations for international migrants and has a growing ethnic minority population. Before the study we searched MEDLINE, Embase, and PsycINFO for epidemiological studies published between Jan 1, 2000 and July 3, 2020, which provided data on absolute and relative rates of suicide among ethnic minority groups. Search terms included: (ethnic minority OR migrant OR refugee OR expatriate OR asylum-seeker OR indigenous OR departee OR foreign born OR foreign worker OR foreign student OR international student, OR minority group OR ethnic group OR BME OR BAME OR CALD OR cultural diversity) AND (suicide OR self-harm) and with no language restrictions. We found that the absolute rate of suicide among ethnic minority populations globally was 12·1 per 100 000 (95% CI 8·4™17·6), with weak statistical evidence of an increased risk of suicide in ethnic minority populations compared with the ethnic majority (rate ratio 1·3 [95% CI 0·9™1·7]). We found considerable heterogeneity between ethnic minority groups and more granular estimates by ethnicity and migrant status are needed. Five studies from England and Wales were identified, providing estimates up until 2007. However, these studies were either restricted to a specific method of suicide or only explored migrant status.

### Added value of this study

We provide suicide rate estimates, for the first time to our knowledge, by 18 ethnic groups, migration, and descendant status using a nationally linked dataset in England and Wales. We found that almost all ethnic minority groups have a lower rate of suicide than the White British majority, with the exception of individuals who identify as being from a Mixed ethnicity background or White Gypsy or Irish Travellers. We show that previous investigations focused on migration status mask an elevated risk of suicide in descendants of migrants (ie, second-generation migrants), and broad ethnic group classifications can lead to some marginalised groups being overlooked (eg, White Gypsy or Irish Travellers).

### Implications of all the available evidence

Differences in suicide risk by ethnicity highlight the importance of tailoring interventions for ethnic minority populations. The two groups highlighted as having an elevated risk of suicide have been overlooked in suicide prevention efforts; therefore, targeted co-produced interventions need to be developed for these groups. Additionally, future research exploring ethnicity and suicide internationally needs to ensure that granular ethnicity classification groups are used; White populations are disaggregated into majority and minority groups; and migrant and descendant populations are identified, as although these groups can be ethnically similar, they can have differing suicide risk profiles. There is a greater clustering of known risk factors for suicide in ethnic minority groups, so the lower-than-expected rate of suicide in most ethnic minority groups requires further attention, as this lower risk might be a consequence of differential misclassification of suicide by ethnicity.

## References

[1] Knipe D, Padmanathan P, Newton-Howes G, Chan LF, Kapur N (2022). Suicide and self-harm. Lancet.

[2] Office for National Statistics (2022). Suicides in England and Wales: 2021 registrations.

[3] Northern Ireland Statistics and Research Agency (2022). Finalised suicide statistics in Northern Ireland, 2015−2021.

[4] Public Health Scotland (2022). Suicide statistics for Scotland.

[5] Office for National Statistics (2021). Mortality from leading causes of death by ethnic group, England and Wales: 2012 to 2019.

[6] Office for National Statistics (2023). Sociodemographic inequalities in suicides in England and Wales: 2011 to 2021.

[7] Ward IL, Finning K, Ayoubkhani D (2024). Sociodemographic inequalities of suicide: a population-based cohort study of adults in England and Wales 2011−21. Eur J Public Health.

[8] Troya MI, Spittal MJ, Pendrous R (2022). Suicide rates amongst individuals from ethnic minority backgrounds: a systematic review and meta-analysis. EClinicalMedicine.

[9] Di Thiene D, Alexanderson K, Tinghög P, Torre La, Mittendorfer-Rutz E (2015). Suicide among first-generation and second-generation immigrants in Sweden: association with labour market marginalisation and morbidity. J Epidemiol Community Health.

[10] Webb RT, Antonsen S, Pedersen CB, Mok PL, Cantor-Graae E, Agerbo E (2016). Attempted suicide and violent criminality among Danish second-generation immigrants according to parental place of origin. Int J Soc Psychiatry.

[11] Office for National Statistics (2022). England and Wales: census 2021.

[12] Zimmerman C, Kiss L, Hossain M (2011). Migration and health: a framework for 21st century policy-making. PLoS Med.

[13] Nafilyan V, Islam N, Mathur R (2021). Ethnic differences in COVID-19 mortality during the first two waves of the coronavirus pandemic: a nationwide cohort study of 29 million adults in England. Eur J Epidemiol.

[14] UK Data Service (2021). Census forms.

[15] Sawyer SM, Azzopardi PS, Wickremarathne D, Patton GC (2018). The age of adolescence. Lancet Child Adolesc Health.

[16] Purdam K, Afkhami R, Crockett A, Olsen W (2007). Religion in the UK: an overview of equality statistics and evidence gaps. J Contemp Relig.

[17] Gearing RE, Alonzo D (2018). Religion and suicide: new findings. J Relig Health.

[18] Kapadia D, Zhang J, Salway S (2022). Ethnic inequalities in healthcare: a rapid evidence review. https://www.nhsrho.org/publications/ethnic-inequalities-in-healthcare-a-rapid-evidence-review/.

[19] Bansal N, Karlsen S, Sashidharan SP, Cohen R, Chew-Graham CA, Malpass A (2022). Understanding ethnic inequalities in mental healthcare in the UK: a meta-ethnography. PLoS Med.

[20] Neeleman J, Mak V, Wessely S (1997). Suicide by age, ethnic group, coroners’ verdicts and country of birth. A three-year survey in inner London. Br J Psychiatry.

[21] Mathur R, Bhaskaran K, Chaturvedi N (2014). Completeness and usability of ethnicity data in UK-based primary care and hospital databases. J Public Health (Oxf).

[22] Harris KL (2018). Biracial American colorism: passing for White. Am Behav Sci.

[23] Harris DR, Sim JJ (2002). Who is multiracial? Assessing the complexity of lived race. Am Sociol Rev.

[24] Brunsma DL, Delgado D, Rockquemore KA (2013). Liminality in the multiracial experience: towards a concept of identity matrix. Identities (Yverdon).

[25] Office for National Statistics (2023). Understanding consistency of ethnicity data recorded in health-related administrative datasets in England: 2011 to 2021.

[26] Campbell ME, Troyer L (2007). The implications of racial misclassification by observers. Am Sociol Rev.

[27] Abdalla S, Quirke B, Fitzpatrick P, Daly L, Kelleher C (2010). All Ireland Traveller health study: our geels. Technical report 2: demography & vital statistics.

[28] The Traveller Movement (2019). Policy briefing addressing mental health and suicide among Gypsy, Roma and Traveller communities in England.

[29] UK Government (2018). Race disparity audit.

[30] Aldridge RW, Nellums LB, Bartlett S (2018). Global patterns of mortality in international migrants: a systematic review and meta-analysis. Lancet.

[31] Urquia ML, Gagnon AJ (2011). Glossary: migration and health. J Epidemiol Community Health.

[32] Office for National Statistics (2020). Provisional long-term international migration estimates (discontinued after August 2020).

[33] Tham SG, Hunt IM, Turnbull P, Appleby L, Kapur N, Knipe D (2023). Suicide among psychiatric patients who migrated to the UK: a national clinical survey. EClinicalMedicine.

[34] Puzo Q, Mehlum L, Qin P (2018). Rates and characteristics of suicide by immigration background in Norway. PLoS One.

[35] Sweeney S, Worrall S (2019). No room at the inn: how easy is it for nomadic Gypsies and Travellers to access primary care?. https://www.gypsy-traveller.org/wp-content/uploads/2019/03/No-room-at-the-inn-findings-from-mystery-shopping-GP-practices.pdf.

[36] Gunnell D, Bennewith O, Simkin S (2013). Time trends in coroners’ use of different verdicts for possible suicides and their impact on officially reported incidence of suicide in England: 1990−2005. Psychol Med.

