# Supplementary material for: Ethnicity and suicide in England and Wales: a national linked cohort study
Source: Lancet Psychiatry. Author manuscript; Available in PMC 2025 Jun 2. (PMC7617724; doi:10.1016/S2215-0366(24)00184-6)
Supplement: Supplementary appendix [file EMS200075-supplement-Supplementary_appendix.pdf]

THE LANCET
Psychiatry

Supplementary appendix

This appendix formed part of the original submission and has been peer reviewed. We post it as supplied by the authors.

Supplement to: Knipe D, Moran P, Howe LD, et al. Ethnicity and suicide in England and Wales: a national linked cohort study. *Lancet Psychiatry* 2024; **11:** 611–19.

Supplementary materials for Ethnicity and suicide in England and Wales: a national linked cohort study by Knipe et al 2024 Lancet Psych

Contents

Supplementary methods 2

Supplementary Table 1 3

Supplementary methods

Secondary suppression

Given the rarity of the outcome suicide and the granularity of the ethnic minority groups presented there are inevitably data cells which have suicide counts below 5. To ensure the non-disclosive nature of the data we have suppressed all cells with counts below 5. All actual zeros exist in the dataset these were also suppressed. The suppression of these zeros would allow for disclosure and therefore we applied secondary suppression to the next smallest figure for the relevant grouping.

Supplementary Table 1 – Proportion of suicide deaths by method, ethnicity and sex.

		N* (%)					
		Poisoning	Hanging	Drowning	Firearms	Jumping	Other
**Male**							
Asian	Bangladeshi	-	13 (32.5)	7 (17.5)	-	-	15 (37.5)
	Chinese	8 (15.7)	20 (39.2)	-	-	6 (11.8)	13 (25.5)
	Indian	58 (16.4)	204 (57.8)	18 (5.1)	-	10 (2.8)	63 (17.8)
	Pakistani	20 (16.9)	57 (48.3)	13 (11.0)	-	5 (4.2)	23 (19.5)
	Other	17 (10.1)	105 (62.5)	6 (3.6)	-	-	36 (21.4)
Black	African	22 (16.5)	48 (36.1)	12 (9.0)	-	5 (3.8)	46 (34.6)
	Caribbean	22 (15.1)	83 (56.8)	-	-	-	34 (23.3)
	Other	7 (15.9)	19 (43.2)	-	-	5 (11.4)	11 (25.0)
Mixed	White/Asian	21 (18.8)	61 (54.5)	-	-	6 (5.4)	19 (17)
	White/Black African	6 (12.2)	28 (57.1)	-	-	-	11 (22.4)
	White/Caribbean	24 (15.8)	94 (61.8)	5 (3.3)	-	7 (4.6)	22 (14.5)
	Other	16 (21.6)	43 (58.1)	-	-	-	7 (9.5)
White	British	3686 (17.7)	12232 (58.6)	731 (3.5)	553 (2.6)	702 (3.4)	2968 (14.2)
	Gypsy Irish Traveller	-	25 (75.8)	-	-	-	-
	Irish	49 (17.1)	146 (50.9)	23 (8.0)	-	10 (3.5)	58 (20.2)
	Other	93 (11.7)	521 (65.5)	17 (2.1)	-	38 (4.8)	123 (15.5)
Arab		-	8 (42.1)	-	-	-	-
Other		13 (18.6)	32 (45.7)	-	-	-	19 (27.1)
**Female**							
Asian	Bangladeshi	-	6 (40.0)	-	-	-	6 (40.0)
	Chinese	13 (23.6)	27 (49.1)	-	-	-	8 (14.5)
	Indian	31 (22.5)	66 (47.8)	12 (8.7)	-	-	25 (18.1)
	Pakistani	9 (23.1)	15 (38.5)	-	-	-	11 (28.2)
	Other	12 (18.2)	39 (59.1)	-	-	6 (9.1)	8 (12.1)
Black	African	19 (33.3)	23 (40.4)	-	-	-	10 (17.5)
	Caribbean	14 (24.6)	26 (45.6)	-	-	-	10 (17.5)
	Other	-	5 (50.0)	-	-	-	-
Mixed	White/Asian	9 (18.4)	25 (51.0)	-	-	-	9 (18.4)
	White/Black African	6 (37.5)	5 (31.3)	-	-	-	-
	White/Caribbean	24 (27.0)	49 (55.1)	-	-	-	11 (12.4)
	Other	9 (29.0)	16 (51.6)	-	-	-	5 (16.1)
White	British	2582 (36.6)	3026 (42.9)	379 (5.4)	13 (0.2)	248 (3.5)	811 (11.5)
	Gypsy Irish Traveller	-	12 (66.7)	-	-	-	-
	Irish	24 (33.8)	25 (35.2)	9 (12.7)	-	5 (7.0)	8 (11.3)
	Other	110 (33.3)	160 (48.5)	10 (3.0)	-	10 (3.0)	40 (12.1)
Arab		-	-	-	-	-	-
Other		-	7 (33.3)	-	-	-	6 (28.6)

Cell counts less than 5 are not presented and secondary suppression has been applied to other cells to avoid statistical disclosure
